# *Hibiscus syriacus* Extract from an Established Cell Culture Stimulates Skin Wound Healing

**DOI:** 10.1155/2017/7932019

**Published:** 2017-11-27

**Authors:** O. di Martino, A. Tito, A. De Lucia, A. Cimmino, F. Cicotti, F. Apone, G. Colucci, V. Calabrò

**Affiliations:** ^1^Dipartimento di Biologia, Università di Napoli Federico II, Complesso Universitario di Monte Sant'Angelo, Via Cintia 4, 80126 Napoli, Italy; ^2^Arterra Bioscience, Via Brin 69, 80142 Napoli, Italy; ^3^Dipartimento di Scienze Chimiche, Università di Napoli Federico II, Complesso Universitario di Monte Sant'Angelo, Via Cintia 4, 80126 Napoli, Italy; ^4^Vitalab srl, Via Brin 69, 80142 Napoli, Italy

## Abstract

Higher plants are the source of a wide array of bioactive compounds that support skin integrity and health.* Hibiscus syriacus*, family Malvaceae, is a plant of Chinese origin known for its antipyretic, anthelmintic, and antifungal properties. The aim of this study was to assess the healing and hydration properties of* H. syriacus* ethanolic extract (HSEE). We established a cell culture from* Hibiscus syriacus* leaves and obtained an ethanol soluble extract from cultured cells. The properties of the extract were tested by gene expression and functional analyses on human fibroblast, keratinocytes, and skin explants. HSEE treatment increased the healing potential of fibroblasts and keratinocytes. Specifically, HSEE significantly stimulated fibronectin and collagen synthesis by 16 and 60%, respectively, while fibroblasts contractility was enhanced by 30%. These results were confirmed on skin explants, where HSEE accelerated the wound healing activity in terms of epithelium formation and fibronectin production. Moreover, HSEE increased the expression of genes involved in skin hydration and homeostasis. Specifically, aquaporin 3 and filaggrin genes were enhanced by 20 and 58%, respectively. Our data show that HSEE contains compounds capable of stimulating expression of biomarkers relevant to skin regeneration and hydration thereby counteracting molecular pathways leading to skin damage and aging.

## 1. Introduction

Wound healing is a dynamic physiological process by which the skin regenerates itself upon injury. The restoration of tissue integrity is the result of the interaction between several distinct cellular elements (keratinocytes, fibroblasts, monocytes/macrophages, and endothelial cells) and extracellular matrix (ECM) components, such as fibronectin and collagen whose contraction encourages the edges of the wound to shrink together [1]. The supply of the ideal microenvironment at the wound surface is fundamental for reaching full skin wound's healing potential. Indeed, adverse factors, such as infection, mechanical stress, or toxic agents, can significantly affect the skin ability to heal. Additionally, the process of wound healing is altered in a dry skin and skin of aged individuals [2]. Skin dryness alters the ability of epithelial cells to migrate and cover the wound site and reduces the supply of white blood cells and nutrients, which are essentials to form new tissues and protect skin against infections [3].

Skin hydration depends on the humidity of the environment and the hygroscopic properties of the stratum corneum, the uppermost epidermal layer. The ability of the stratum corneum to retain water relies on the natural moisturizing factor (NMF), a set of substances including ions, small solutes, and free amino acids which are largely formed by the breakdown products of filaggrin protein [4].

Skin care products are often developed from plants. Higher plants are the source of a wide array of biochemicals that can support the health and integrity of the skin and are widely used in cosmetic formulations.

*H. syriacus*, family Malvaceae, is a plant of Chinese origin already known in Asia for its antipyretic, anthelmintic, and antifungal properties [5].* H. syriacus* extract was previously shown to have antioxidant capacity [6] and antiproliferative effects on human lung cancer cells [7]. However, the leaves of Hibiscus genus are traditionally acclaimed as hair tonic in the Indian system of medicine. Accordingly, topical application of* H. syriacus* extract was found to stimulate hair growth thereby validating the ethnomedical use of this plants for hair loss treatment [8].

Remarkably,* H. rosa-sinensis* of the genus* Hibiscus* was previously reported to efficiently act as wound healing agent by increasing cellular proliferation and collagen synthesis [9]. More recent* in vitro* and* in vivo* studies indicated* Hibiscus syriacus* L. flower absolute (HSF) as a highly effective agent for wound treatment because of its ability to promote keratinocyte proliferation and migration [10].

A chemical study performed on raw material from the epigeal part of the plant revealed the presence of flavonoids [dihydroquercetin, herbacetin, kaempferol, saponaretin, and saponarin] previously demonstrated to be able to reduce UVB-induced erythema and tumorigenesis [11]. Furthermore,* H. syriacus* extract was also found to be rich in anthocyanins, fatty acids, and several types of pigments [12].

Nowadays, cell suspension cultures from plants provide a viable alternative over whole plant cultivation for the production of secondary metabolites. They represent standardized, contaminant-free, and biosustainable sources of beneficial bioactive compounds for cosmetics on an industrial scale [12, 13].

We have established stem cell suspension cultures of* H. syriacus* and prepared a hydro/alcoholic extract rich in flavonoids and coumarins. Here, we present a study aimed at evaluating the wound healing and hydration properties of* Hibiscus syriacus* extract from cell suspension culture.

## 2. Materials and Methods

### 2.1. Establishment of Plant Cell Suspension Cultures

*Hibiscus syriacus* leaves (cultivated in Campania region, Italy) were surface-sterilized using 70% EtOH for 15 min and 1% bleach. After sterilization, the leaf explants were each cut into 0.5 cm pieces, wounded, and placed on solid Gamborg B5 medium (Duchefa Biochemie), containing 500 mg L^−1^ myoinositol, 30 g L^−1^ sucrose, 1 mg L^−1^ of 2,4-dichlorophenoxyacetic acid (2,4-D), 0.1 mg L^−1^ kinetin, 1 mg L^−1^ adenine, and 7.5 mg L^−1^ plant agar, pH 5.7 [14]. Friable calluses were obtained after 5 weeks of cultivation in the dark at 27°C. To initiate stem cell suspension cultures, 50 mg of 40- to 45-day-old calluses was resuspended in 25–30 mL of liquid medium Gamborg B5 (the same as the one used for callus growth but without agar) and incubated at 27°C in the dark under constant orbital stirring (110 rpm) in 2 L volume Erlenmeyer flasks.

### 2.2. Extract Preparation from Plant Cell Cultures

*Hibiscus syriacus* stem cells (500 g) were harvested, tested for microbial contamination, and then frozen at −30°C or −80°C until processing. 200 ml of EtOH 96% was added. The mix was stirred for 1 h at RT and filtered and the alcohol-soluble fraction was collected in a sterile container placed in an ice bath. The ethanol was eliminated by using the Rotavapor until 1/10 of the extract was obtained. The obtained dried ethanolic extract was analyzed by TLC in different solvent mixtures:* i*-PrOH : H_2_O 8 : 2, v/v; CHCl_3_ :* i-*PrOH 9 : 1, v/v; EtOAc : *n*-hexane* i*-PrOH 6 : 4, v/v. Analytical and preparative TLC were performed on silica gel (Kieselgel 60, *F*_254_, 0.20 and 0.5 mm, resp.) and on reversed phase (Kieselgel 60 RP-18, *F*_254_, 0.20 mm) plates (Merck, Darmstadt, Germany). The spots were visualized by exposure to UV light, or by spraying first with 10% H_2_SO_4_ in MeOH and then with 5% phosphomolybdic acid in EtOH, followed by heating at 110°C for 10′. The EtOAc organic extract residue (20.0 mg) was purified by preparative TLC on silica gel using as eluent CHCl_3_ :* i*-PrOH 95 : 5, v/v, yielding eight homogeneous fractions. ^1^H NMR spectra were recorded at 400 or 500 MHz in CDCl_3_ on Bruker (Karlsruhe, Germany) and Varian (Palo Alto, CA, USA). The same solvent was used as internal standard.

### 2.3. Skin Cell Cultures

Spontaneously immortalized human HaCaT keratinocytes and Human Dermal Fibroblasts (HDF) were maintained in DMEM supplemented with 10% fetal bovine serum (FBS) and 5%  CO_2_ humidified atmosphere at 37°C as previously described [14, 15].

### 2.4. MTT Assay

HDF or HaCaT cells, plated into 96-well plates at a density of 1.3 × 10^3^ cells/well, were grown for 12 h and treated for 48 h with different amounts of the* H. syriacus* extract (% v/v). After treatment, cells were washed with PBS and incubated with 100 *μ*L per well of reaction buffer (10 mM Hepes, 1.3 mM CaCl_2_, 1 mM MgSO_4_, 5 mM glucose, and 0.5 mg/mL of MTT 3-[4,5-dimethylthiazol-2-yl]-2,5-diphenyl tetrazoliumbromide) in PBS buffer at pH 7.4. After 3 h, at 37°C and 5%  CO_2_, cells were solubilized in 100 *μ*L of solubilization solution (Triton X-100, 0.1 N HCl in 10% isopropanol), and the plate was incubated for 4 h at RT. The developed colour was quantified at 595 nm by a multiwell-plate reader Victor3 (Perkin Elmer, Waltham, MA, USA) [16].

### 2.5. Gene Expression Analysis

Total RNA was extracted from HaCaT cells with the GenElute Mammalian Total RNA Purification Kit (Sigma-Aldrich, Milano, Italy) and treated with DNAse I at 37°C for 30′ [16]. Reverse transcription was performed using the RevertAid™ First Strand cDNA Synthesis Kit (Thermo Fisher Scientific, Dallas, TX, USA). RT-PCR was performed with the Quantum RNA™ kit (Ambion) containing primers to amplify 18S rRNA along with competimers that reduce the amplified 18S rRNA product within the range to allow it to be used as endogenous standard. The primers and PCR conditions were designed as in Table S1. The PCR products were visualized on 1.5% agarose gel and quantified with the Geliance 200 Imaging system (Perkin Elmer). The obtained amplicons were normalized to the 18S band and reported as percentage of untreated controls (fixed as 100%). RT-PCRs reactions were made in triplicate, and the results of representative experiments were reported in the graphs [17].

### 2.6. ELISA Assay

HDF cells were seeded at a density of 9 × 10^3^ in 96-well plates and treated with HSEE. Ascorbic acid (300 *μ*M) and TGF *β* (2.5 ng/mL) were used as positive control for pro-collagen I and fibronectin production, respectively. After the treatments, the medium was removed and the cells were washed with PBS 1x, fixed in paraformaldehyde [PFA] 4% for 10′, washed three times with PBS 1x, and permeabilized with 1% Triton X-100 in PBS for 30′. The cells were then treated for 30′ with 0.5% Tween and 5% BSA in PBS and incubated a 4°C with primary goat polyclonal antibody, raised against human fibronectin (C-20) or pro-collagen type I (Y-18) (Santa Cruz Biotechnology, Dallas, TX, USA), diluted 1 : 500 in PBS 1x, containing 0.5% Tween and 1% BSA. 16 h later, samples were washed 3 times with PBS + 0.5% Tween and incubated with anti-goat secondary antibody, labeled with Horse-Radish Peroxidase (HRP) (Santa Cruz Biotechnology) and diluted 1 : 1000 in PBS 1x, containing 0.5% Tween and 1% BSA. One hour later, plates were washed 3 times with PBS 1x and the amount of fibronectin or pro-collagen type I produced by the cells was measured by a colorimetric reaction, using a 0.5 mg/ml solution of o-phenylendiamine (OPD) (Sigma-Aldrich) 0.012%  H_2_O_2_ in citrate buffer 50 mM. The plate was incubated at RT for 60′ until the yellow colour developed. The absorbance of each sample was measured at 490 nm by a Multiwell Plate Reader Victor3 (Perkin Elmer).

### 2.7. Wound Healing Assay

HaCaT or HDF cells, seeded at density of 1 × 10^6^ in 6-well plates, were incubated until reaching 100% confluence. Same areas of each well were displaced by scratching a line through the cell layer by a pipet tip, simulating a wound. Floating cells were removed by PBS washing. Media containing 0.5% FBS with or without treatment were added to the cells. Mitomycin C (10 *μ*g/ml) was included in the media for HDF treatment to prevent cell proliferation. To estimate the relative migration of the cells, the unclosed cell-free areas at times 0 and 7 or 24 h after treatments were compared, for each condition, by using the software Image J. Means of left and right wound margins were calculated [18].

### 2.8. Collagen Contraction Assay

The collagen contraction assay was carried out according to Ngo et al. method [19]. 2 × 10^5^ HDF cells were seeded in 24-well plate mixed with 2 mg/ml of collagen solution. The plate was incubated at 37°C for 30′ to induce collagen gel formation. The medium with treatment was then added and the plate was incubated for the night. After 24 h, the gel was released from the bottom of the well to induce collagen gel contraction. Pictures were taken 24 hours after the release. To estimate the relative collagen contraction of the cells, the gel areas at times 0 and 24 h after treatments were compared, for each condition, by using the software Image J.

### 2.9. Cell Cycle Analysis

Flow cytometry analysis was performed as previously described [20]. Briefly, cells seeded at a density of 2.5 × 10^5^ were plated in 35 mm dishes and treated with HSEE (from 0.0004% to 0.1%). 24 h later, cells were washed twice with PBS and harvested at 1500*g* with 0.05% trypsin in 0.15%  Na_2_EDTA. Cells were then centrifuged, washed in PBS, fixed with ice-cold methanol (100%), and left for 20′ on ice. Cells were washed in PBS and then incubated with propidium iodide (50 *μ*g/ml) and RNAse A (10 *μ*g/ml) for 30′ at RT in the dark. Data acquisition was performed using a CyAn ADP Flow Cytometer (Beckman Coulter, Inc., Milano, Italy) and Summit Software.

### 2.10. Tests on Skin Explants

The ex vivo tests were performed by Laboratoire BIO-EC, Longjumeau, Ile-de-France, France (S1), on 24 explants coming from an abdominal plasty of a 35-year-old female patient. The explants were kept alive in a BEM culture medium (BIO-EC's Explants Medium) at 37°C in a humid, 5%  CO_2_ atmosphere. Two mechanical lesions of 3.5 mm in diameter were made on the 18 explants by a biopsy punch (Day 0), and after 1 day (D1) 6 explants were left untreated and 6 were treated each with 0.002% HSEE and 6 with 100 ng/mL TGF-*β*1, as positive control. The compounds were applied topically as solutions and spread by using a small spatula. The control explants did not receive any treatment. The capacity of the compounds to stimulate wound healing activity was evaluated by microscopic observation after 3 days (D3), by measuring the morphology of the reconstituted epidermis and the fibronectin production. In parallel, the remaining 6 explants, not treated and unwounded, were monitored for the same time period in order to guarantee the goodness of the explants during the preservation procedures and the test evolution. The collected explants were cut in two parts: half was fixed in buffered formalin and half was frozen at −80°C to be used for fibronectin analysis. After fixation for 24 hours, the samples were dehydrated and impregnated in paraffin using a Leica TP 1010 dehydration automat (Leica, Wetzlar, Germany). Five *μ*m thick sections were made using a Leica RM 2125 Minot-type microtome, mounted on Superfrost® histological glass slides and then stained according to Masson's trichrome, Goldner variant [21]. The microscopical observations were made using an Olympus BX43 microscope and the pictures digitized by a numeric DP72 Olympus camera with CellD storing software. From the observation of 24 section pictures for each treatment (4 for each sample), the length of the newly synthesized epidermis was measured and the average values were calculated in *μ*m. Fibronectin immunostaining was performed on frozen sections with a monoclonal anti-fibronectin antibody (Santa Cruz Biotechnology) diluted at 1 : 200 in PBS-BSA 0.3% and 0.05% Tween 20, for 1 h at RT with a biotin/streptavidin amplifier system, and revealed using FITC (Thermo Fisher Scientific, Waltham, MA, USA). The nuclei were poststained with propidium iodide. 24 pictures for each treatment were taken and fluorescence intensity was measured by ImageJ software. The values obtained as number of pixels were converted in percentage with respect to untreated controls, arbitrarily set as 100%.

### 2.11. Statistical Analysis

Unpaired *t*-test was performed using GraphPad software. Quantitative data were presented as mean ± standard deviation (SD). Comparison between data was analyzed using *t*-tests. Differences were considered statistically significant when *P* values were less than 0.05 (*P* < 0.05).

## 3. Results and Discussion 

### 3.1. HSEE Chemical Characterization

A* H. syriacus* cell suspension culture was used for the preparation of an ethanolic extract (HSEE). By thin layer chromatography (TLC) of the HSEE, we observed the presence of hydrophilic low and high molecular weight compounds. Glucose was identified as the main sugar, by comparison with an authentic standard, together with a mixture of unknown saccharides. The ethanolic extract (1.0 g) was exhaustively extracted with EtOAc giving a solid residue (2%). TLC investigation of this extract showed the presence of at least eight LMW lipophilic compounds, while ^1^H NMR analysis revealed the presence of aromatic signals, methoxy groups, and double bounds probably belonging to flavonoids and coumarins (Figure S1).

Although not exhaustive, chemical analysis of our extract from* H. syriacus* cell suspension culture showed that, beside the large amount of monosaccharides, including glucose and a mixture of oligosaccharides, the organic extract contained a mixture of unknown low molecular weight metabolites. Preliminary ^1^H NMR investigation showed the presence of signals significant for the presence of flavonoids, coumarins already known to have prohealing properties [22] and naphthalene carbaldehyde. Further analysis is needed in order to identify all the compounds contained in the extract.

### 3.2. HSEE Improves the Wound Healing Process

To determine the right usage concentrations, we evaluated HSEE cytotoxicity. HaCaT keratinocytes were treated for 48 h with increasing amount of HSEE (from 0.0004 to 0.05% v/v) and cell viability was determined by MTT assay. No significant cytotoxicity was recorded at all concentrations tested (Figure S1).

**Figure 1:**
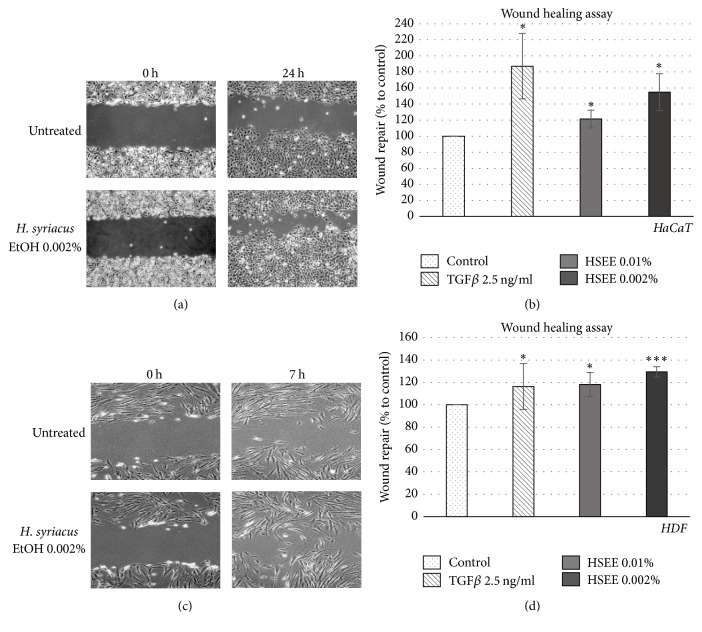
Effect of* Hibiscus syriacus* ethanolic extract (HSEE) on wound healing. (a) Confluent HaCaT cells were wounded and then treated with HSEE at the indicated concentrations into the 0.5% FBS-containing medium. TGF *β* at concentration of 2.5 ng/ml was used as positive control. At times 0 and 24 h, phase-contrast pictures of the wounds at four different locations were taken. (b) The relative migration of the cells was expressed as % of wound repair compared to control. Each column value represents the average of three experiments and error bars indicate standard deviations. *P* value < 0.05 is represented by *∗*. (c) Confluent HDF cells were wounded and then treated with HSEE at indicated concentrations for 7 h in 0.5% FBS-containing medium. TGF*β* at concentration of 2.5 ng/ml was used as positive control. Mitomycin C (10 *μ*g/ml) was included in the media to prevent cell proliferation. At times 0 and 7 h, phase-contrast pictures of the wounds at four different locations were taken. (d) For each condition, the unclosed cell-free areas at time 0 were compared to those at 7 h by using the software Image J. The relative migration of the cells was expressed as % of wound repair compared to control. Each column value represents the average of three experiments and error bars indicate standard deviations.* P* value < 0.05 is represented by *∗*. *P* value < 0.001 is represented by *∗∗∗*.

Next, we evaluated the ability of HSEE in promoting wound healing. Fibroblasts and keratinocytes are key players of the wound healing process in the skin; therefore confluent cell monolayers of either HDF or HaCaT keratinocytes were mechanically wounded and the closure of the wound edges in the presence of 0.002 and 0.01% (v/v) of HSEE was evaluated by light microscopy. Wound healing in the presence of Transforming Growth Factor *β* (TGF *β*), known to induce a faster healing rate, was used as positive control [23]. As shown in Figure 1(a), compared to the untreated control, treatment of HaCaT cells with 0.002% HSEE for 24 hrs significantly improved the wound healing response. The wound repair in 0.002 and 0.01% HSEE increased by 50 and 20%, respectively (Figure 1(b)). Similarly, the treatment with 0.002% HSEE increased by 30% the wound healing capacity in HDF, after only 7 h of treatment (Figures 1(c) and 1(d)).

Both cell proliferation and migration contribute to reepithelization of wounded skin. To evaluate the contribution of cell proliferation to HSEE-induced wound closure, we analyzed the effect of HSEE on cell cycle distribution by flow cytometry. HaCaT cells treated with different doses of HSEE for 24 h were labeled with propidium iodide and analyzed by flow cytometry. We observed no significant changes on cell cycle distribution except for a slight increase in G1 cells at 0.002% HSEE indicating that HSEE stimulates cell migration rather than proliferation (Figure S1).

### 3.3. HSEE Induces the Synthesis of Extracellular Matrix Components

**Figure 2:**
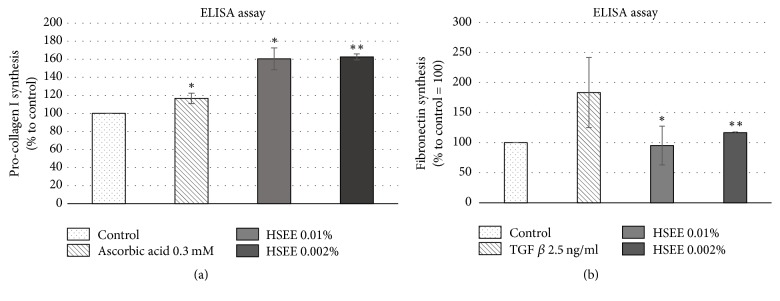
Effect of* Hibiscus syriacus* ethanolic extracts (HSEE) on pro-collagen I and fibronectin production. (a) HDF cells were seeded in 96-well plates at a density of 1.5 × 10^4^ per well and treated with HSEE at the indicated concentrations for 24 h. Each experimental condition was done in quadruplicate. Ascorbic acid at concentration of 300 *μ*M was used as positive control. Each column value represents the average of three experiments and error bars indicate standard deviations. *P* value < 0.05 is represented by *∗*. *P* value < 0.01 is represented by *∗∗*. (b) HDF cells were seeded in 96-well plates at a density of 9 × 10^3^ per well and treated with HSEE at the indicated concentrations for 72 h. Each experimental condition was done in quadruplicate. TGF *β* at concentration of 2.5 ng/mL was used as a positive control. Each column value represents the average of four experiments and error bars indicate standard deviations. *P* value < 0.05 is represented by *∗*. *P* value < 0.01 is represented by *∗∗*.

To understand whether the effect of HSEE on wound healing was also linked to an increase of ECM protein synthesis, we measured the production of fibronectin and pro-collagen I in HDF cells treated with HSEE, by Elisa assay. Induction by ascorbic acid or TGF*β* was used as positive control for pro-collagen type I and fibronectin, respectively. Under HSEE stimulation, pro-collagen type I increased by 60% at both concentrations tested (Figure 2(a)) while fibronectin increased by 16% at the lower HSEE concentration (Figure 2(b)). Similar to integrins, fibronectin binds extracellular matrix components, such as collagen, thereby playing a major role in cell adhesion and migration. Thus, induction of both fibronectin and collagen constitutes a key element in tissue regeneration and can be associated with a higher migration ability of fibroblast.

### 3.4. HSEE Improves Collagen Contraction

**Figure 3:**
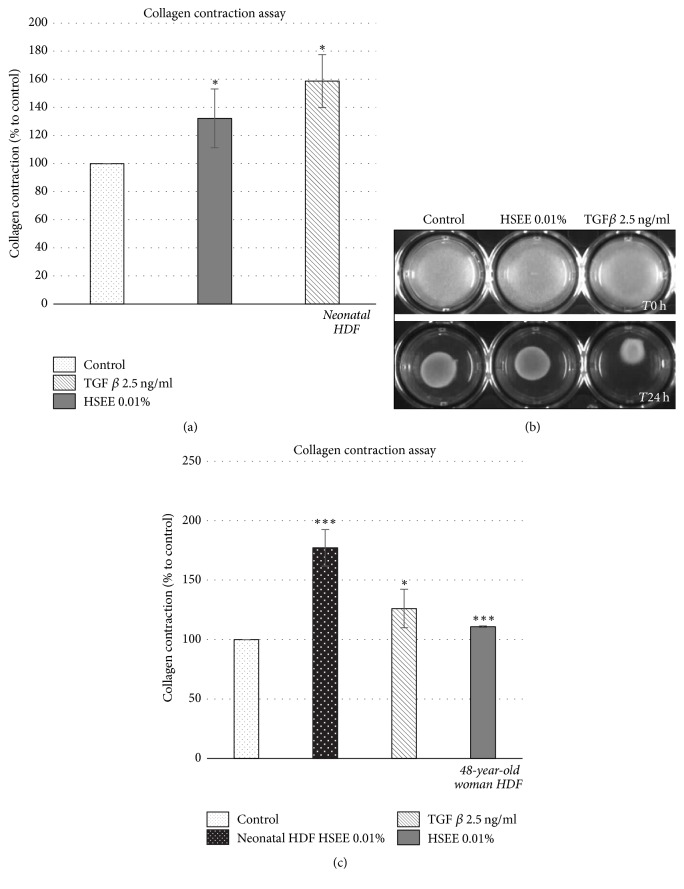
Effect of* Hibiscus syriacus* ethanolic extract (HSEE) on collagen contraction. (a–c) 2 × 10^5^ HDF cells were seeded in 24-well plates mixed with 2 mg/ml of collagen solution. The plates were incubated at 37°C for 30′ to induce collagen gel formation. TGF*β* at concentration of 2.5 ng/ml was used as positive control. At the end of incubation the medium with treatment was added and the plates were incubated overnight. The day after the gel was released from the bottom of the well to induce collagen gel contraction. 24 h later, pictures were taken and gel area was estimated. Each column value of the plot represents the average of four experiments and error bars indicate standard deviations. *P* value < 0.05 is represented by *∗*.* P* value < 0.001 is represented by *∗∗∗*. (b) Representative image of collagen floating gel after 24 hours of HSEE treatment. HDF cells treated with HSEE showed higher contractibility than untreated HDF cells.

Fibroblasts play a critical role in wound healing by generating forces for wound contraction. The effect of HSEE treatment on fibroblast contractility was evaluated by 3D-collagen gel contraction assay using neonatal HDF (Figures 3(a) and 3(b)) and fibroblasts from a 48-year-old female donor (Figure 3(c)). The obtained results proved that HSEE at 0.01% improved neonatal HDF wound contraction by 30% (Figure 3(a)). On the other hand, HDF from a 48-year-old female donor that have a collagen contractile strength 70% lighter than neonatal cells increased the wound contraction by 10% upon treatment with HSEE at 0.01% (Figure 3(c)). Therefore, HSEE treatment can stimulate wound healing by increasing the contraction capacity of fibroblasts, deriving from either young or aged skin.

### 3.5. HSEE Induces Filaggrin and Aquaporin 3 Gene Expression

Hydration supports the wound healing process as it improves the ability of epithelial cells to migrate and cover the wound site. To analyze the potential hydration properties of* H. syriacus*, we measured the effect of HSEE extract on aquaporin 3 (AQP3) and filaggrin (FLG) gene expression. Aquaporin 3 (AQP3) is an integral membrane pore protein expressed more in the basal than in the upper layer of epidermis. AQP3 selectively conducts water molecules in and out of the cell and prevents the passage of ions and other solutes [4, 24]. Filaggrin, instead, is a filament-associated protein that binds to keratin fibers and is responsible for the integrity and water-proofing capacity of the upper layer of the skin [25]. HaCaT cells were treated with 0.002 or 0.01% HSEE for 6 h. Total RNA was extracted and subjected to semiquantitative RT-PCR to determine the level of FLG and AQP3 specific transcripts. Retinoic acid treatment was used as positive control [26].

**Figure 4:**
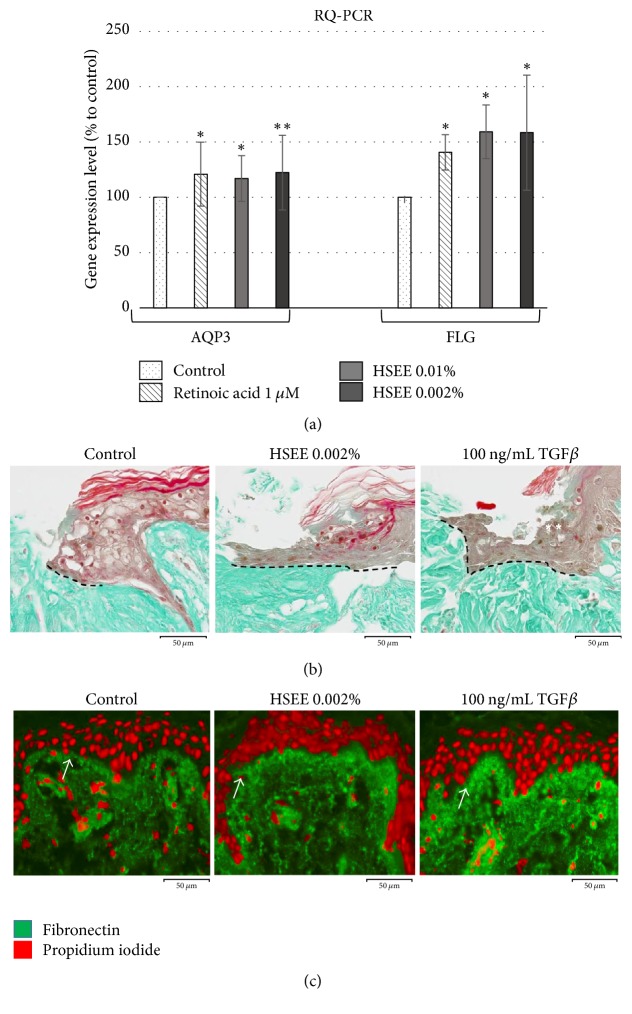
AQP3 and FLG gene expression analysis in HaCaT cells treated with HSEE and HSEE effect on ex vivo skin explants. (a) HaCaT cells were seeded at concentration of 1.5 × 10^5^ cells in 35 mm dishes. After 24 h, cells were treated for 6 h with HSEE at the indicated concentrations. Total RNA was prepared and reverse-transcribed. Obtained cDNAs were used to evaluate AQP3 and FLG gene expression by performing a semiquantitative RT-PCR. Retinoic acid at concentration of 1 *μ*M was used as positive control. The values were normalized to rRNA 18S. Each column value represents the average of four experiments and error bars indicate standard deviations. *P* value < 0.05 is represented by *∗*; *P* value < 0.01 is represented by *∗∗*. *P* value < 0.001 is represented by *∗∗*. (b) Representative experiment showing the effect of HSEE on ex vivo skin explants. The samples were wounded and treated with HSEE and TGF*β* as indicated. After 3 days, the explants were processed to produce sections, which were then stained for morphology analysis. (c) Fibronectin was detected by immunostaining using a monoclonal primary antibody (Santa Cruz Biotechnology) and FITC-conjugated secondary antibody (green). The nuclei were poststained with propidium iodide (red). The length of the newly generated epidermis is indicated by the dotted lines. The leading edge is indicated by arrows.

As shown in Figure 4(a), compared to untreated control, HSEE treatment increased AQP3 and FLG expression by 20% and 60%, respectively. Proteolytic fragments of FLG contribute to the natural moisturizing factor (NMF) that is essential for stratum corneum hydration. By increasing FLG expression, HSEE may contribute to NMF production and maintenance of skin water balance.

### 3.6. Wound Healing Assay on Human Skin Explants

Tissue explants are a skin model that more accurately mimic the* in vivo* environment. Human skin explants coming from an abdominal biopsy were kept in a nutrient medium and subjected to a biopsy punch to produce a cut of 3.5 mm diameter. After 24 h, some explants were left untreated as control; others were treated topically, either with 0.002% HSEE or with 100 ng/mL TGF*β*. After 3 days, the samples were processed for microscopic analysis in order to measure the wound recovery capacity and the amount of fibronectin produced. The treatment with HSEE improved the skin regeneration capacity by 38%, while the growth factor TGF*β* produced an increase of 21%. A representative example of this assay is shown in Figure 4(b). The length of the newly generated epidermis (indicated by the dotted lines) was 152.5 ± 12.4 *μ*m for the untreated control, 210.9 ± 28.8 *μ*m for the HSEE treated samples, and 183.9 ± 16.4 *μ*m for TGF*β* (*P* value < 0.01). Furthermore, analysis of the fibronectin expression by immunostaining revealed that in samples treated with HSEE the amount of fibronectin synthesized during the healing process was 19.4 ± 6.7% (Figure 4(c)). This value was higher than that of untreated controls and comparable to that observed in TGF*β* treated samples (25.1 ± 10.2%; *P* value < 0.05) thereby confirming the data obtained by the* in vitro* studies.

## 4. Conclusions

Our study clearly indicates that HSEE extract from cell suspension culture enhances the healing potential of the skin by accelerating the wound healing process and stimulating the expression of biomarkers involved in skin regeneration and hydration. Moreover, our ex vivo studies, in human injured skin biopsies, clearly show that HSEE topical treatment significantly improves the skin wound closure by inducing an increase of neoepidermis. Altogether, these results suggest that HSSS is a valuable bioactive compound to use in cosmetic industry and alternative medicine.

## Abbreviations

ECM: Extracellular matrix
RT: Room temperature.

## Supplementary Materials

Supplementary Materials Table S1: Gene specific primers used. Figure S1: 1H NMR spectrum of the EtOAc organic extract of H. syriacus cell culture extract. Aromatic signals, methoxy groups, and double bounds probably belonging to flavonoids and coumarins are indicated. Figure S2: Analysis of cell viability in HaCaT cells after treatment with HSEE. Figure S3: Cell cycle profiling of HaCaT cells treated with Hibiscus syriacus.
