# Supplementary material for: Hibiscus syriacus Extract from an Established Cell Culture Stimulates Skin Wound Healing
Source: Biomed Res Int. 2017 Nov 27;2017:7932019. doi: 10.1155/2017/7932019 (PMC5733167; doi:10.1155/2017/7932019)
Supplement: Supplementary Materials — Table S1: Gene specific primers used. Figure S1: 1H NMR spectrum of the EtOAc organic extract of H. syriacus cell culture extract. Aromatic signals, methoxy groups, and double bounds probably belonging to flavonoids and coumarins are indicated. Figure S2: Analysis of cell viability in HaCaT cells after treatment with HSEE. Figure S3: Cell cycle profiling of HaCaT cells treated with Hibiscus syriacus. [file 7932019.f1.pdf]

Supporting Information

Table S1.

Gene Specific Primers used:

AQP3-FW: *5'GATCAAGCTGCCCATCTA 3'*

AQP3-RV: *5'TGGGCCAGCTTCACATTCT 3'*

FLG-FW: *5' AGAGCTGAAGGAACTTCTGG 3'*

FLG-RV: *5' GTGTCATAGGCTTCATCC 3'*

The amplification reactions were performed as follows: 2' at 94°C followed by 35 cycles of 94°C for 30'', annealing temperature (AQP3 = 60°C FLG = 58°C) for 30s, and 72°C for 30-60'', with a 10' final extension at 72°C.

Fig. S1. 1H NMR spectrum of the EtOAc organic extract of *H. syriacus* cell culture extract. Aromatic signals, methoxy groups and double bounds probably belonging to flavonoids and coumarins are indicated.

Fig. S2. Analysis of cell viability in HaCaT cells after treatment with HSEE. HaCaT keratinocytes were seeded into 96-well plates at a density of $1.3{\times}10^3$ cells/well, grown for 8 hrs and treated for 48h with different concentrations of the extracts (from 0.05% w/v to 0.0004% w/v). After treatments, the cell viability was measured by the MTT assay. The values are means of five independent measures obtained from one representative experiment among three, and the P-value < 0.05 is represented by *; P-value < 0.01 is represented by **.

Fig. S3. Cell cycle profiling of HaCaT cells treated with *Hibiscus syriacus*.

(A) DNA cell cycle distribution of HaCaT cells treated with increasing amount of HSEE (0.0004 to 0.1% v/v) determined by FACSscan analysis. $2x10^5$ HaCaT cells were treated with increasing amount of HSSE. After 24 h, the cells were harvested and their DNA cell cycle distributions were determined. (B) Plots representing the percentage of HaCaT cells in each phase of the cell cycle.

di Martino et al. Figure 1

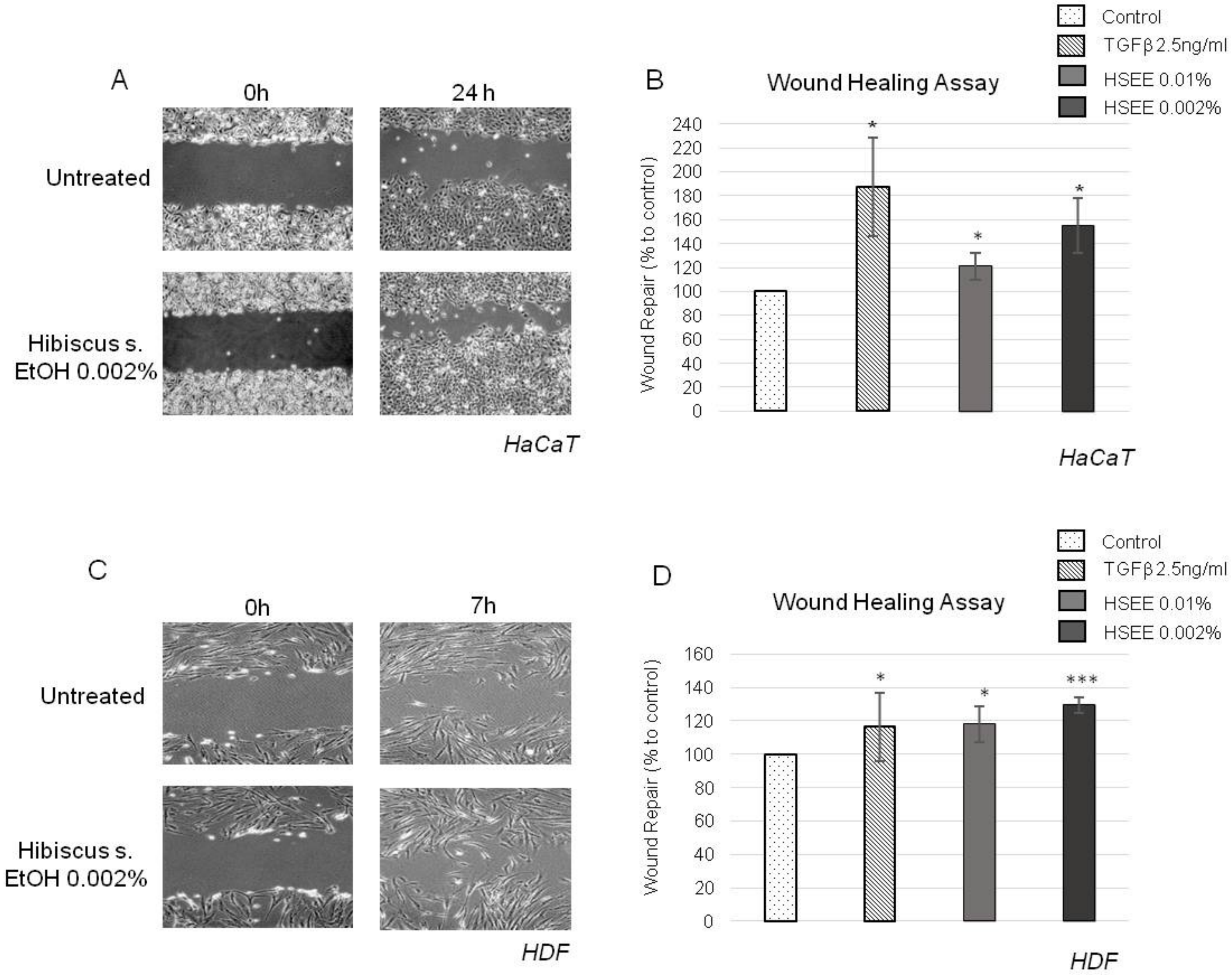

di Martino et al. Figure 2

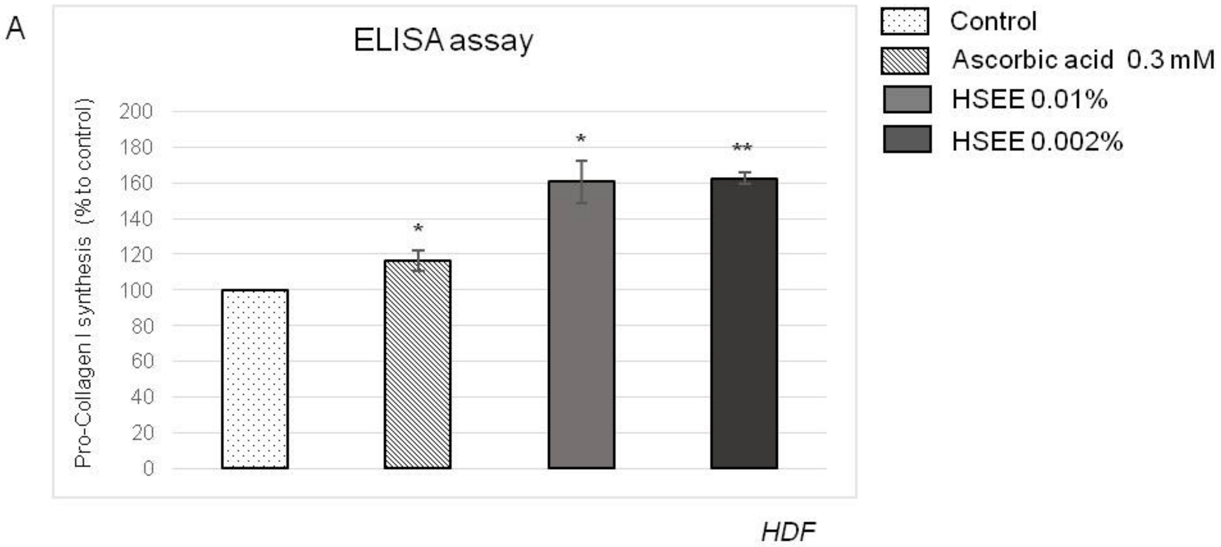

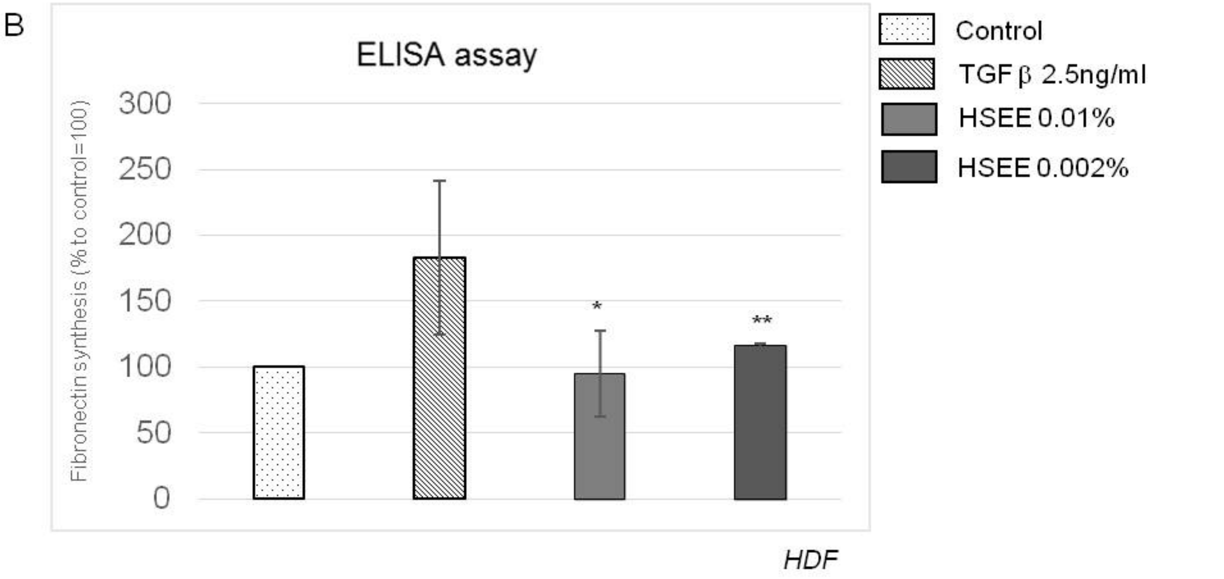

di Martino et al. Figure 3

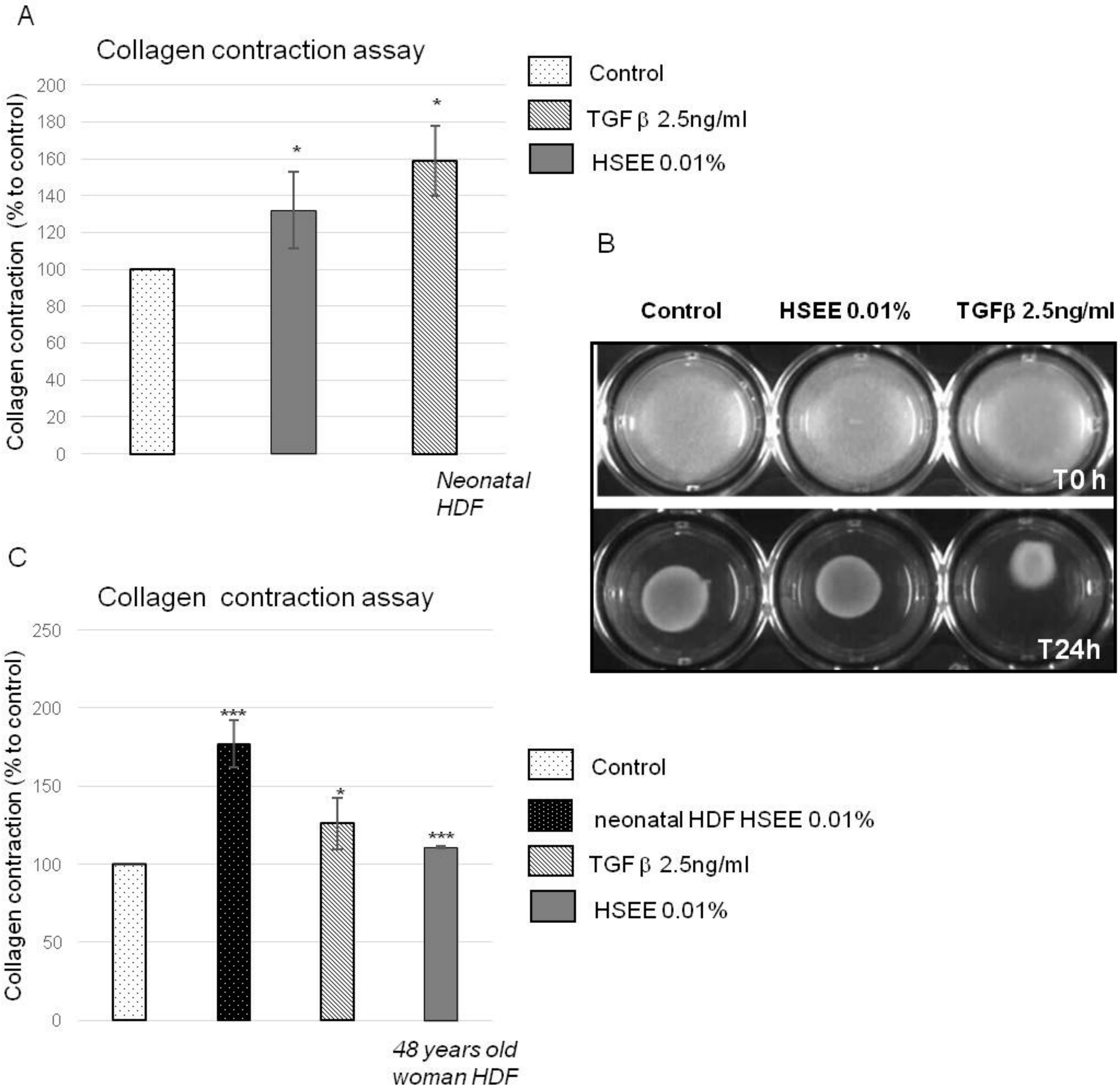

di Martino et al. Figure 4

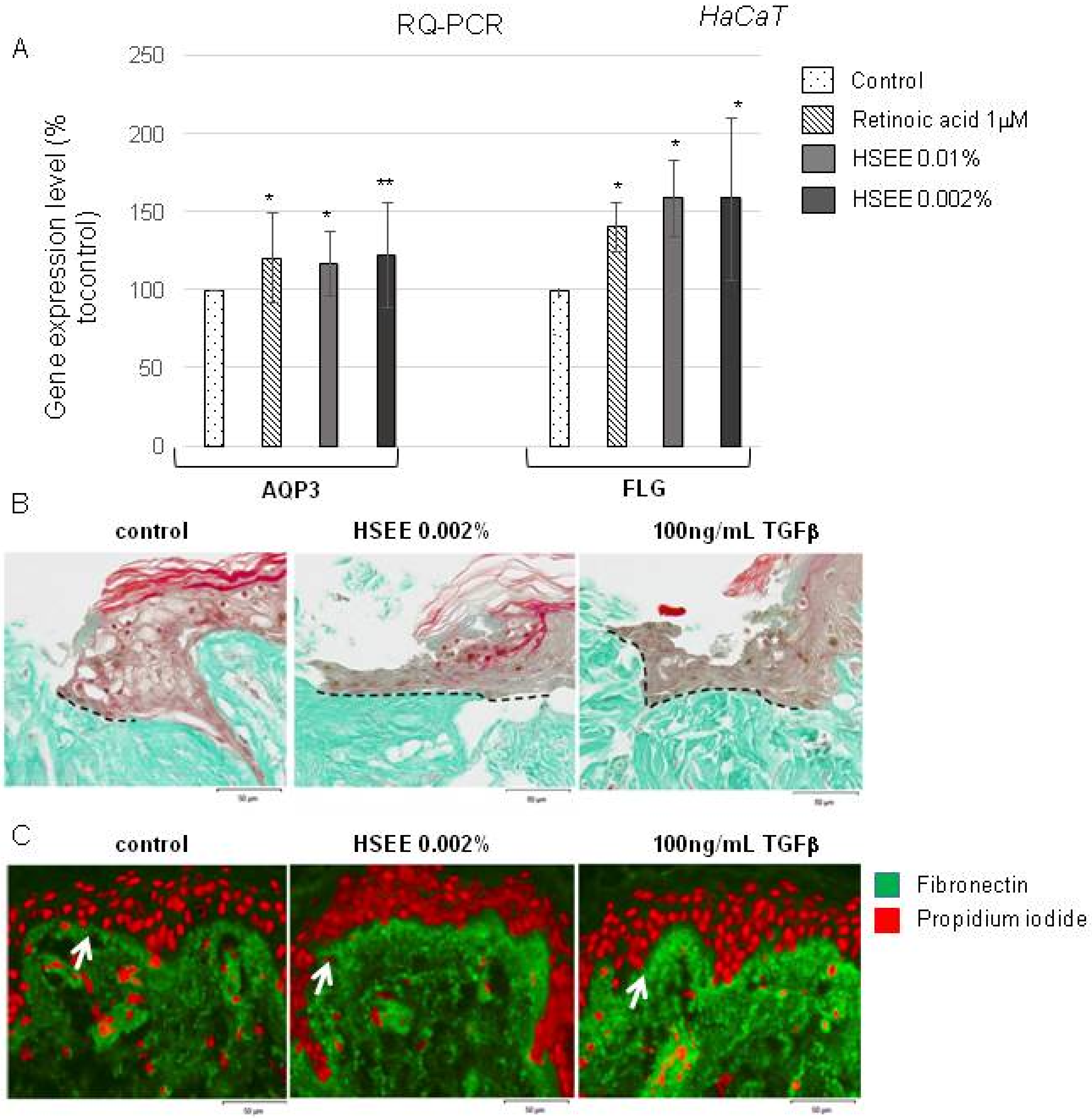

Figure S.1

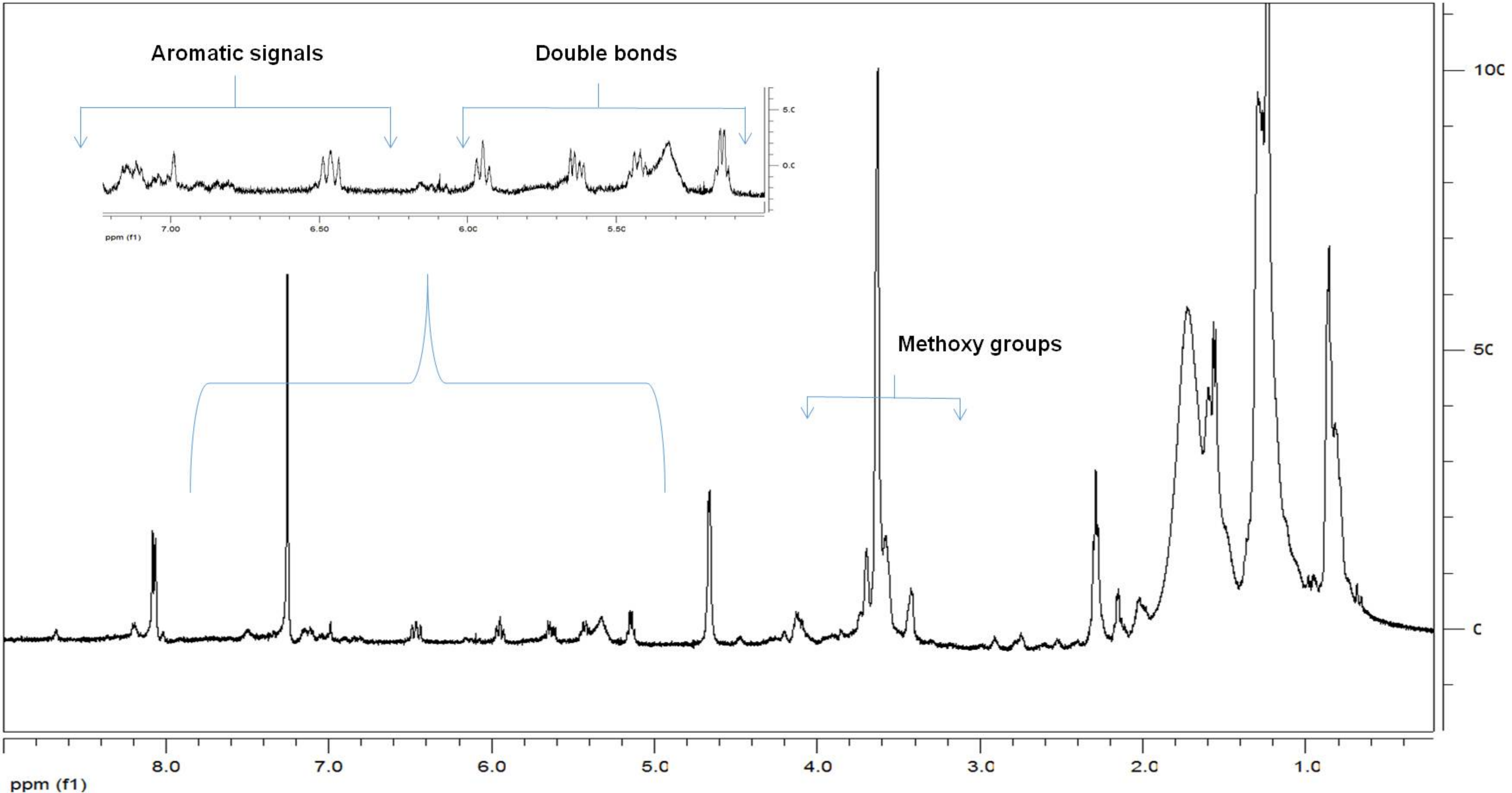

Aromatic signals
Double bonds
Methoxy groups
ppm (f1)
8.0
7.0
6.0
5.0
4.0
3.0
2.0
1.0
100
50
0
7.00
6.50
6.00
5.50
5.0
0.0

Figure S.2

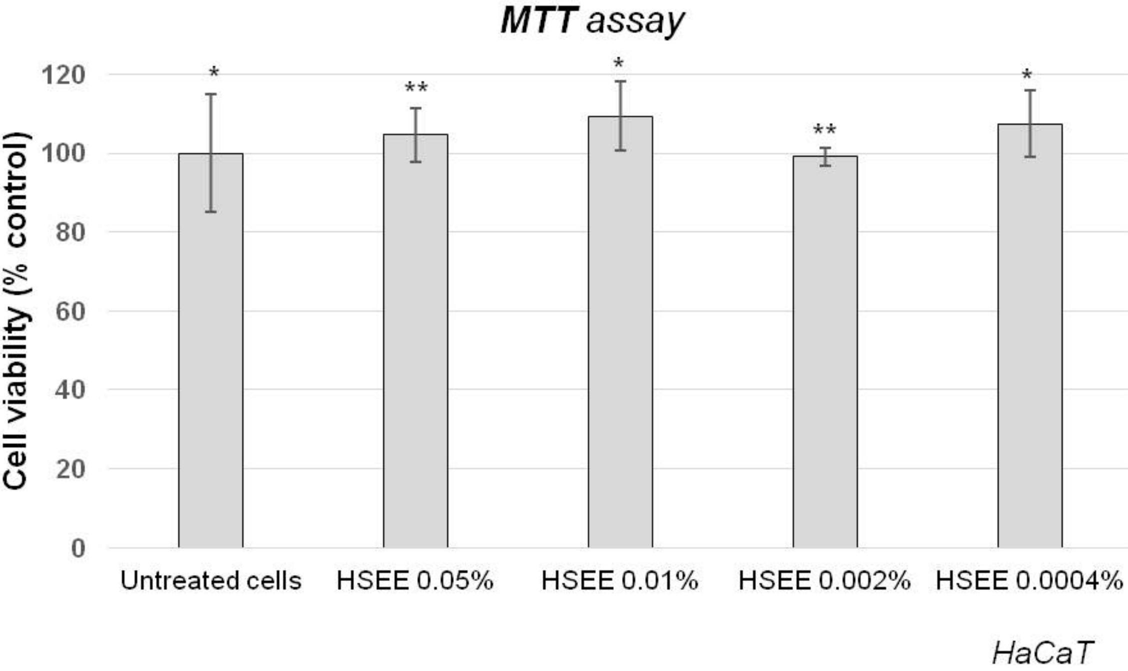
MTT assay
Cell viability (% control)
120
100
80
60
40
20
0
*
**
*
**
*
Untreated cells
HSEE 0.05%
HSEE 0.01%
HSEE 0.002%
HSEE 0.0004%
HaCaT

di martino et al. Figure S.3

A

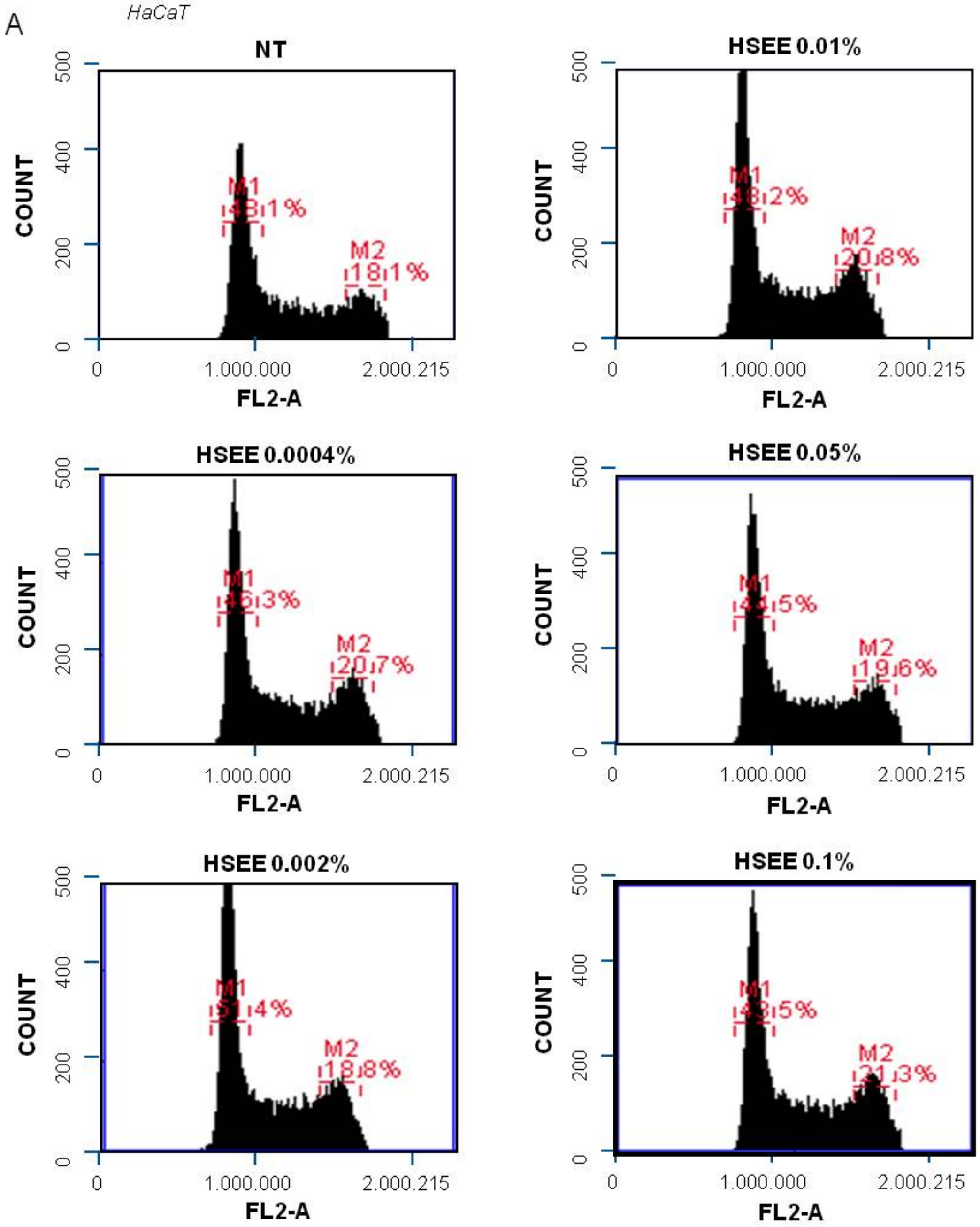

B

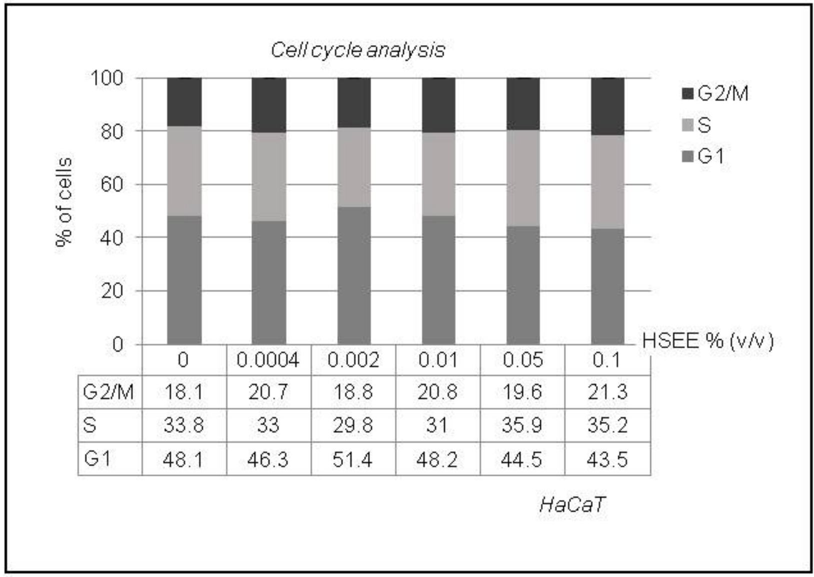

	0	0.0004	0.002	0.01	0.05	0.1
G2/M	18.1	20.7	18.8	20.8	19.6	21.3
S	33.8	33	29.8	31	35.9	35.2
G1	48.1	46.3	51.4	48.2	44.5	43.5

Supporting Information

Gene Specific Primers used:

AQP3-FW: *5'GATCAAGCTGCCCATCTA 3'*

AQP3-RV: *5'TGGGCCAGCTTCACATTCT 3'*

FLG-FW: *5' AGAGCTGAAGGAACTTCTGG 3'*

FLG-RV: *5' GTGTCATAGGCTTCATCC 3'*

The amplification reactions were performed as follows: 2' at 94°C followed by 35 cycles of 94°C for 30'', annealing temperature (AQP3 = 60°C FLG = 58°C) for 30s, and 72°C for 30-60'', with a 10' final extension at 72°C.
